# Use of different endpoints to determine the bioavailability of polychlorinated dibenzo-*p*-dioxins/furans (PCDD/Fs) and polychlorinated biphenyls (PCBs) in Sprague–Dawley rats

**DOI:** 10.1038/s41598-022-25042-3

**Published:** 2022-11-28

**Authors:** Haitao Shen, Jianlong Han, Rongfa Guan, Delei Cai, Yibin Zheng, Zhen Meng, Qing Chen, Jingguang Li, Yongning Wu

**Affiliations:** 1 Zhejiang Provincial Center for Disease Control and Prevention, 3399 Binsheng Road, Hangzhou, 310051 China; 2 College of Food Science and Technology, Zhejiang University of Technology, Hangzhou, 310014 China; 3 NHC Key Laboratory Food Safety Risk Assessment, Chinese Academy of Medical Sciences Research Unit (2019RU014 Food Safety), China National Center for Food Safety Risk Assessment, Building 2, Guangqu Road 37, Beijing, 100022 China

**Keywords:** Environmental sciences, Risk factors

## Abstract

Liver, fat (adipose tissue), blood, and feces are common endpoints used to determine the bioavailability of persistent organic pollutants (POPs). However, it is not known whether the bioavailability of each endpoints is comparable or whether there is a comprehensive endpoint that can be used for all congeners for the measurement of bioavailability. In this study, we observed the accumulation and distribution of 10 polychlorinated dibenzo-*p*-dioxins/furans (PCDD/Fs) and 18 polychlorinated biphenyls (PCBs) in different organs of Sprague–Dawley (SD) rats and calculated the bioavailability based on feces, liver, and fat endpoints. Our results indicated that PCB 126, PCB 169, and 50% of PCDD/F congeners were mainly accumulated in the liver, with a bioavailability ranging from 28.9 to 50.6%. On the other hand, higher chlorinated (> 5 Cl) PCB congeners were mainly accumulated in adipose tissues, with a bioavailability ranging from 20.1 to 82.2%, while lower chlorinated (< 5 Cl) pollutants, such as 2,3,7,8-TeCDF, 2,3,7,8-TeCDD, 1,2,3,7,8-PeCDF, and PCB 28, 52, 77, 81, were likely metabolized over 36% in rats during the 8-week experimental period. If we considered metabolization (degradation) as a type of bioavailable process, then the fecal endpoint was a feasible option. However, if we considered the selective accumulation behavior of some congeners in different organs/tissues, then there was no single comprehensive endpoint suitable for all congeners. Lastly, female rats showed significantly higher PCDD bioavailability than male rats at low dose level (0.2 ng/100 g b.w./d); however, the difference in PCB bioavailability between female and male rats was not significant.

## Introduction

Polychlorinated dibenzo-*p*-dioxins/furans (PCDD/Fs, also known as dioxin) and polychlorinated biphenyls (PCBs) are typical persistent organic pollutants (POPs). These contaminants can travel long distances through air and water, bio-accumulate in the food chain, and accumulate in higher animals, including humans. These pollutants are associated with adverse effects, including cancer, cardiovascular diseases, diabetes, hormone disruptions, learning disabilities, neurological ailments, obesity, and infertility^1–3^. When quantifying the exposure of humans to POPs for risk assessment calculations, it is assumed that pollutants in the matrix (food, soil, and water) are 100% soluble in the gastrointestinal tract and 100% absorbable by the systemic circulation. However, only a fraction of the pollutants is bioavailable, and as such, this assumption overestimates the real exposure dose. Therefore, the measurement of bioavailability is critical to reduce the uncertainty of risk assessment.

The term bioavailability (or absolute bioavailability) generally refers to a fraction of the external dose, usually by ingestion, that reaches the circulation of mammals. In pharmacokinetic studies, bioavailability describes the rate and degree of drug entering the blood circulation, which is calculated by the area under the drug time curve (AUC). In the 1990s, the concept of bioavailability was introduced into the field of risk assessment to assess the health risks of mammals to environmental pollutants, but there is considerable ambiguity in the specific definition, especially how to calculate this value^4^. For example, some researchers use the traditional AUC method to calculate the bioavailability of benzopyrene (BAP) or polychlorinated biphenyl (PCB) in soil^5^, while other researchers express the bioavailability using a ratio of the concentration in a specific target organ (liver, for example) to the intake concentration, which has been reported in studies of perfluorooctanoic acid (PFOA)^6^ and heavy metals such as lead^7^. However, data obtained by different methods cannot be compared.

To determine the bioavailability of POP, in vivo assays using different animal models (mice, rats, and swine), as well as different bioavailability endpoints (muscles, organs, blood, adipose tissues, DNA adducts, or enzyme activation), have been carried out. The advantages and disadvantages of each model and endpoint have been previously summarized in the literature^4^. However, because of the reported differences in the three-dimensional structure and the unknown physiological mechanism of POP, the distribution of POP congeners in different organs and tissues varies widely. For example, when using the Fischer 344 male rat model, penta-chlorodibenzofuran (PeCDF) is rapidly removed from the systemic circulation and accumulates in the liver and adipose tissues. Three days after administration, 70% of the intravenous dose of PeCDF was detected in the liver, 7% in the fat, 1% in the skin, and 0.5% in the muscle^8^. In this case, fat or muscle can be selected as the endpoint, although this may be greatly inconsistent with the results of the liver endpoint. Another study reported that the relative bioavailability did not significantly vary among the three endpoints (adipose tissue, liver, and kidneys) in mice^9^. However, the contaminants only consisted of non-dioxin-like PCBs (NDL-PCBs), and dioxin-like PCBs (DL-PCBs) and PCDD/Fs, which could accumulate in specific organs, were not observed. For example, researchers reported that the toxic equivalent (TEQ) concentration of DL-PCB in wild roe deer liver (21.66 ± 15.58 pg WHO_2005-TEQ_ g^−1^ fat) was tenfold higher than that in adipose tissue (2.05 ± 1.28 pg WHO_2005-TEQ_ g^−1^ fat), but the concentration of NDL-PCB in the liver (21.15 ± 19.05 ng g^−1^ fat) was only two-fold higher than that in adipose tissue (9.37 ± 10.12 pg g^−1^ fat)^10^, which suggested that DL-PCBs were more prone to accumulate in the liver. In our recent study, we demonstrated that the most toxic coplanar congeners, PCB 126 and PCB 169, were more abundant in the liver than in any other organ in the rat, pig, and fish. Therefore, as far as bioavailability is concerned, when using different organs or tissues as the endpoints, inconsistent observations among these endpoints might lead to the underestimation of the real exposure dose of certain congeners. Furthermore, bioavailability in terms of pharmacokinetics focuses on the rate and degree of a certain drug entering the systemic circulation. For some lipophilic exogenous pollutants, the redistribution rate from blood to fat or organs is very fast (accumulation effect), so the concentration in the blood might not be an ideal indicator to represent the toxic effect. It is also difficult to observe dose–response relationships. Moreover, the calculation of bioavailability based on specific organs depends on the accurate acquisition of the mass of all organs. However, in practical in vivo settings, it is difficult to obtain the accurate mass of some tissues such as blood and muscle. In addition, the results in specific organs do not represent the bioavailability of the whole organism.

The bioavailability of POP, if measured using feces as the endpoint, might avoid these shortcomings. It considers the entire body, instead of specific organs, as the depot for POP to calculate the bioavailability. The concentrations of POPs determined in feces represent the non-absorbable or non-bioavailable fraction of the administered dose^4^, while the dose in each organ is the bioavailable fraction. Some studies using feces as the endpoint have been conducted to investigate the bioaccumulation of PCDD/Fs^8,11,12^, NDL-PCBs^13,14^, and heavy metals^15,16^. The main aims of this study were: (1) to report the bioavailability of 10 toxic PCDD/Fs, 12 DL-PCBs, and 6 NDL-PCBs using feces, liver, and fat tissues as the endpoints respectively; and (2) to compare the differences in the bioavailability among these endpoints to clarify whether feces can be used as an indirect endpoint for bioavailability measurement.

## Materials and methods

### Standard solutions and chemical reagents

Native dioxin/furan mixtures (M-8280A-PAK/M-8280B-PAK, containing 10 PCDD/F congeners), native NDL-PCB mixtures (PCB-DUTCH7-SET), and native DL-PCB (12 individual standard solutions), which were used for the fortification of soybean oil, were purchased from AccuStandard (New Haven, CT, USA). ^13^C_12_-labeled PCDD/F compounds, which were used as surrogates (EDF-8999) and internal standards (EDF-5999) for PCDD/Fs, were purchased from Cambridge Isotope Laboratories (Tewksbury, MA, USA). ^13^C_12_-labeled PCB compounds, which were used as surrogates (WP-LCS and EC-9605 SS) and internal standards (EC-9605 RS) for PCBs, were obtained from Wellington Laboratories (Guelph, ON, Canada). Pre-mixed PCDD/F and PCB calibration standards were purchased from Cambridge Isotope Laboratories and Wellington Laboratories, respectively.

All solvents were pesticide grade and purchased from Duksan Pure Chemicals (Ansan, Kyungkido, South Korea). Aluminum oxide (neutral, 70–200 mesh) and florisil (60–100 mesh) were purchased from Sigma-Aldrich (Shanghai, China). Silica gel 60 (70–230 mesh) was purchased from Merck (Shanghai, China).

### Animal model

#### Sprague–Dawley rats and group designations

The in vivo experiment was conducted using Sprague–Dawley (SD) rats in stainless steel metabolic cages over the course of 8-weeks. The SD rats were allocated into three groups, namely, control group, low dose group, and high dose group. Each group was consisted of three male (♂) and three female (♀) rats. Therefore, there were six rats in each group, and a total of 18 rats were studied. Specific-pathogen-free SD rats were provided by Shanghai Laboratory Animal Co. Ltd. The protocol was approved by the Animal Care Committee of Zhejiang Provincial Center for Disease Control and Prevention (ZJCDC), performed in compliance with ZJCDC Animal Care guidelines, and carried out in compliance with the ARRIVE guidelines (Animal Research: Reporting of In Vivo Experiments).

#### Administration dose

Rats had free access to water and food obtained from the Zhejiang Experimental Animal Center. Rats were weighed on a balance and treated with soybean oil at a constant volume/body weight ratio (0.2 mL/100 g b.w.) each day. The concentrations of PCB/PCDD/F spiked into the soybean oil were 0 ng/mL (control group), 1 ng/mL for PCB (low dose group), 0.2 ng/mL for PCDD/F (low dose group), 5 ng/mL for PCB (high dose group), and 1 ng/mL for PCDD/F (high dose group). The body weight and volume of administration were recorded for each rat for subsequent intake calculations. All rats were fasted for 24 h before treatment.

#### Excrement collection and sample preparation

Urine and feces were collected in individually-labelled vessels and bags each day and then stored at − 20 °C. Eight weeks later, the rats were anesthetized by isoflurane and sacrificed to obtain the liver, blood, muscle, fat, and other organs (including kidneys, pancreas, testes or ovaries, and heart), which were stored at  − 40 °C. The urine and feces produced by the same rat during this period were combined into 18 pooled urine mixtures and 18 pooled feces mixtures. The feces were freeze-dried, homogenized into powder, and weighed. A 5-g aliquot of each feces mixture and a 20-mL aliquot of urine mixture were used for chemical analysis.

### Sample extraction, cleanup, and instrumental analysis

Feces, rodent feeds, and other rat bio-samples were freeze-dried for over 24 h before accelerated solvent extraction (ASE) with 1:1 (v/v) hexane in dichloromethane. For blood and urine samples, we used hydrochloric acid digestion, followed by liquid–liquid extraction (LLE) with 1:1 (v/v) hexane in dichloromethane. The cleanup procedure is described in our previous study with minor modifications^17^. Briefly, the feces extract in ~ 4 mL of hexane was transferred completely onto a gel permeation chromatography (GPC) system (for the other samples, this step was omitted). Next, the concentrated extract was subjected sequentially to multilayer silica gel and basic alumina chromatography columns for further cleanup. Finally, the extracts were loaded onto a self-prepared florisil column to separate PCBs from PCDD/Fs. The fractions were evaporated to ~ 40 μL under a weak stream of nitrogen gas. Before analysis, 1,000 pg of each internal standard was added to each sample. The quantification by HRGC-HRMS used an Agilent 6890 gas chromatograph (Agilent Technologies, Santa Clara, CA, USA) coupled with an Autospec Ultima mass spectrometer (Micromass, Manchester, UK) operating in an EI mode of 35 eV and a trap current of 600 μA. A 1-μL aliquot of the sample was injected to a split-less injector onto a DB-5 MS fused silica capillary column (60 m×250 μm I.D., film thickness 0.25 μm, J&W Scientific, USA) with helium as carrier gas at a constant flow rate of 1.0 ml min^−1^ for PCDD/Fs and 1.4 ml min^−1^ for PCBs. The oven temperature program for PCDD/Fs was as follows: start at 120 °C, held for 1 min, increased to 220 °C at 7.5 °C min^−1^ and held for 15 min, increased to 260 °C at 5 °C min^−1^ and held for 7 min, and finally increased to 310 °C at 5 °C min^−1^ and held for 1 min. For PCBs, the program started at 80 °C and held for 2 min, increased to 150 °C at 15 °C min^−1^, increased to 270 °C at 3 °C min^−1^ and held for 3 min, and finally increased to 310 °C at 15 °C min^−1^ and held for 1.5 min. Quantification was performed using an isotope dilution method.

### QA/QC

The QA/QC procedure was similar to our previous study^18^. Prior to performing the in vivo assay, the rodent feed and soybean oil used as the dosing vehicle were screened to determine the background concentrations of all PCDD/Fs/PCBs. After PCDD/Fs/PCBs were spiked into the soybean oil, the high and low spiked soybean oil samples were reanalyzed to confirm the spiking concentrations. The samples were analyzed in batches of twelve. Each batch consists of one procedure blank, eight real samples and one real sample repetition, one solvent blank, and one mid-level calibration standard (CS3). A certified reference material consisting of fish tissue (WMF-01, Wellington Laboratory Inc.) was extracted, processed, and analyzed with every 2 batches to verify the method accuracy.

In this study, 90% of our surrogate recoveries ranged from 40 to 120%, and none of the surrogate recoveries were outside of the quality control acceptance criteria range (25–150%)^18^. In addition, all concentrations of PCBs/PCDD/Fs in the WMF-01used in this study were within an acceptable range. We participated the 21st round of the Norwegian Interlaboratory Study on POPs in Food and achieved reliable results (lab code: L-35, Z scores range: − 0.5 ~ 1.1, data are available at: https://www.fhi.no/globalassets/dokumenterfiler/rapporter/2021/interlaboratory-comparison-on-pops-in-food-2020_rapport-2021.pdf).

### Bioavailability calculations

The absolute bioavailability (%) of the PCDD/F and PCB were calculated as follows:

1 $${\text{Congener}}\,{\text{bioavailability}} \left( {{\text{tissue}}/{\text{organ endpoint}}, \% } \right) = \frac{{{\text{POPs amount in tissue}}/{\text{organ}}}}{{{\text{administrated}}\,{\text{amount}} }} \times 100$$

2 $$ {\text{Congener }}\,{\text{bioavailability }}\left( {{\text{fecal}}\,{\text{endpoint}},{\text{ \% }}} \right) = \left( {1 - \frac{{{\text{POPs }}\,{\text{amount}}\,{\text{ in}}\,{\text{ feces}}}}{{{\text{administrated }}\,{\text{amount }}}}} \right) \times 100 $$

3 $${\text{TEQ }}\,bioavailability \left( \% \right) = \frac{{\mathop \sum \nolimits_{{{\text{i}} = 1}}^{{10{\text{ or }}12}} {\text{TEF*}}\left( {{\text{input}} - {\text{output}}} \right)}}{{\mathop \sum \nolimits_{{{\text{i}} = 1}}^{{10{\text{ or }}12}} {\text{TEF*input}}}} \times 100$$

where in Eq. (1), “POPs amount in tissue/organ” is the total amount of pollutants accumulated in special target tissue/organ, such as fat and liver, which equals the concentration of pollutants in each target tissue/organ multiplied by the corresponding organ mass (gram, Table S2); while “administrated amount” is the total gavage dose, which equals the concentration of POPs in the low or high dose group (ng/mL, Table S1) multiplied by the total gavage volume (mL, Table S1). Where in Eq. (2), “POPs amount in feces” is the total amount (mass weight, gram) of PCDD/F or PCB congener detected in the rat excrement (level of POP in urine was negligible, < 0.01%), while “administered amount” is the same as that described in Eq. (1). Where in Eq. (3), TEQ (toxic equivalent) bioavailability was defined as the weighed sum of each congener (g) multiplied by their corresponding toxic equivalency factor (TEF), “input” referred to the total gavage dose, and “output” was the total amount (mass weight, gram) of PCDD/F or PCB congener detected in the rat excrement (mainly in feces). There were 10 toxic congeners for PCDD/Fs and 12 toxic congeners for dioxin-like PCBs observed in the present study.

For the control group, the bioavailability of these pollutants was calculated using the amounts pre-existing in the soybean oil and rodent chow. For low and high dose groups, the pre-existing concentrations in the soybean oil and rodent chow were far below (< 0.1%) than the spiking level, and the bioavailability of PCB/PCDD/F was calculated using only spiking amounts. A complete list of all PCBs/PCDD/Fs used in this study, including pre-existing concentrations in the soybean oil and rodent chow, dose amounts, and feces sample amounts, is presented in Table S1-S4.

### Statistical analysis

Excel 2016 was used for statistical analyses. The bioavailability of PCDD/Fs and PCBs was expressed as mean ± standard deviation. Values of some congeners smaller than the detection limit were regarded as zero for statistical calculations. Pearson’s correlation coefficients were used to test the correlations between gender and bioavailability. The statistical difference was considered significant if *p* ≤ 0.05.

## Results and discussion

**Figure 1:**
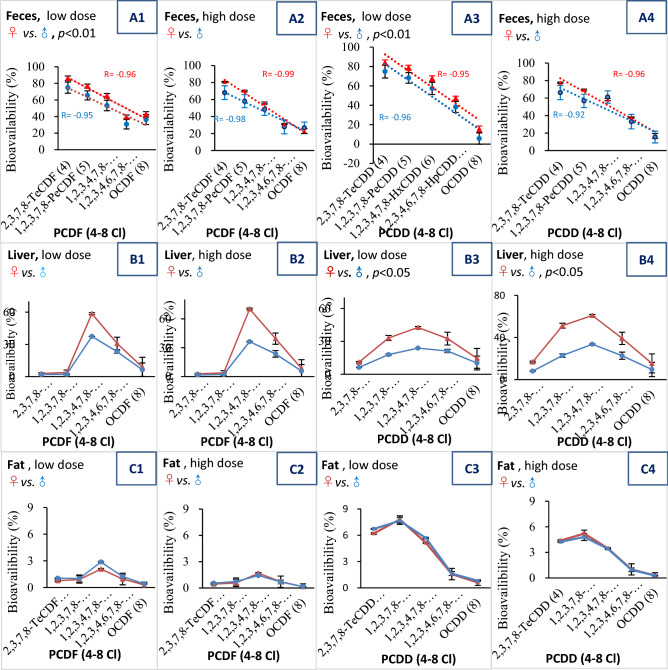
PCDD/F bioavailability measured by different endpoints. Female (♀) data were presented in red and male (♂) data in blue. R: Pearson's correlation coefficient; For PCDD/F: low dose: 0.2 ng/100 g b.w./d; high dose: 1 ng/100 g b.w./d.

**Figure 2:**
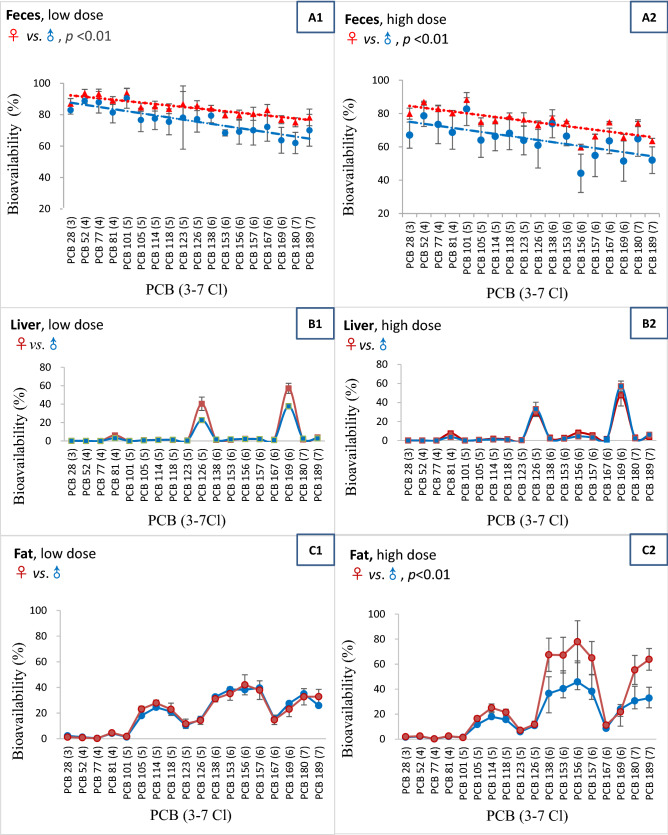
PCB congener bioavailability measured by different endpoints. Female (♀) data were presented in red and male (♂) data in blue. R: Pearson's correlation coefficient; For PCBs: Low dose: 1 ng /100 g b.w./d; High dose: 5 ng/100 g b.w./d.

In this study, 10 toxic PCDD/F congeners and 12 dioxin-like PCB (DL-PCB) congeners were examined. For the convenience of risk assessment, as well as to present the results in a concise way, we introduced the concept of TEQ (toxic equivalent) bioavailability as defined by Eq. (3). The TEQ bioavailability of PCDD/Fs and PCBs is summarized in Table 2. Furthermore, congener specific bioavailability is illustrated in Figs. 1 and 2, and additional details are available in Table S1-S4.

### Is feces a feasible endpoint to determine POP bioavailability?

The primary aim of this study was to determine whether excrement, mainly feces, can be used as a comprehensive endpoint for the measurement of PCDD/F and PCB bioavailability. We measured the concentration of PCDD/F and PCB in excrement (urine, feces), blood, fat, liver, muscle, and other organs (including kidneys, pancreas, testes or ovaries, and heart) and observed that the bioaccumulation percentage of these pollutants was either at a negligible level in urine (< 0.01%) and blood (~ 0.03%) or at a low level in muscle (~ 1.8%) and other organs (0.7–5.6%) (Table S4). On the contrary, feces, liver, and fat tissues were the main PCDD/F and PCB depots in rats, and thus, in this study, we mainly examined the bioavailability of these congeners based on feces, liver, and fat endpoints, which were calculated using Eqs. (1) and (2). As summarized in Table 1, the PCDD/F bioavailability (%) under different endpoints varied in the range of 13.3–77.4 (mean: 52.3 ± 8.6, *feces*), 2.3–50.6 (mean: 21.6 ± 7.3, *liver*), and 0.8–20.6 (mean: 7.3 ± 2.0, *fat*), while the PCB bioavailability (%) varied in the range of 63.2–88.8 (mean: 75.1 ± 9.6, *feces*), 0.0–49.9 (mean: 6.1 ± 1.7, *liver*), and 0.4–82.2 (mean: 34.9 ± 11.1, *fat*), respectively. Budinsky et al. observed the oral bioavailability of dioxins and furans in rats^19^. After oral administration with corn oil for 30 days, using the liver or fat endpoint, the bioavailability of 2,3,7,8-TeCDD (liver: 13.9%/10.5%; fat: 31.9%/17.4%), 1,2,3,7,8-PeCDD (liver: 26.5%/31.3%; fat: 25.0%/20.6%), 1,2,3,4,6,7,8-HpCDD (liver: 26.5%/28.9%; fat: 4.1%/4.2%), 2,3,7,8-TeCDF (liver: 7.2%/2.3%; fat: 5.5%/2.3%), and 1,2,3,4,7,8-HxCDF (liver: 54.5%/50.6%; fat: 5.5%/6.5%) was generally comparable to the results of this study, which used the liver or fat endpoint (in parentheses, reference value/this study value). In another study, Wittepe et al. reported the mean bioavailability of P_(4–8)_CDD/F in the liver (19.0 ± 13.2%) and fat (12.6 ± 10.2%) of young Goettingen minipigs^11^, which was also similar to the results of this study (liver: 21.6 ± 7.3%; fat: 7.3 ± 2.0%). These similarities from different studies showed that when the liver or fat was used as the endpoint, the results were reproducible.

**Table 1:** PCDD/F/PCB congener bioavailability obtained by different endpoints (mean ± std).

Congeners	Bioavailability (%)			Speculated metabolization ratio	Congeners	Bioavailability (%)			Speculated metabolization ratio
	Feces	Liver	Fat*			Feces	Liver	Fat*	
2,3,7,8-TeCDF	77.4 ± 9.0	2.3 ± 0.7	2.3 ± 0.8	74%	PCB 28	79.2 ± 8.8	0.1 ± 0.0	2.8 ± 0.7	76%
1,2,3,7,8-PeCDF	67.5 ± 8.8	3.0 ± 1.0	2.6 ± 0.7	62%	PCB 52	87.0 ± 6.4	0.1 ± 0.0	2.7 ± 1.3	84%
1,2,3,4,7,8-HxCDF	**55.6 ± 8.1**	**50.6 ± 16.3**	**6.5 ± 2.0**	0	PCB 101	88.8 ± 5.0	0.1 ± 0.0	2.4 ± 0.5	86%
1,2,3,4,6,7,8-HpCDF	**32.4 ± 5.8**	**29.3 ± 8.2**	**2.9 ± 0.9**	0	PCB 138	**78.6 ± 10.8**	**2.0 ± 1.0**	**67.7 ± 28.8**	9%
OCDF	32.1 ± 8.5	7.4 ± 1.9	0.8 ± 0.5	24%	PCB 153	**72.5 ± 7.7**	**2.0 ± 0.8**	**73.0 ± 24.4**	0
2,3,7,8-TeCDD	75.7 ± 8.4	10.5 ± 4.4	17.4 ± 4.1	48%	PCB 180	**69.0 ± 8.5**	**2.4 ± 1.0**	**62.0 ± 19.3**	5%
1,2,3,7,8-PeCDD	**68.3 ± 9.7**	**31.3 ± 13.7**	**20.6 ± 5.0**	16%	PCB 77	84.4 ± 8.7	0.0 ± 0.0	0.4 ± 0.1	84%
1,2,3,4,7,8-HxCDD	**57.5 ± 15.4**	**40.3 ± 15.5**	**14.3 ± 3.6**	3%	PCB 81	79.8 ± 9.2	5.1 ± 2.2	5.5 ± 2.1	69%
1,2,3,4,6,7,8-HpCDD	**39.1 ± 6.2**	**28.9 ± 8.6**	**4.2 ± 1.1**	6%	PCB 105	75.1 ± 9.6	0.7 ± 0.2	28.0 ± 7.5	46%
OCDD	**13.3 ± 6.1**	**12.4 ± 3.4**	**1.6 ± 1.1**	0	PCB 114	76.2 ± 8.9	1.3 ± 0.4	38.5 ± 7.4	36%
					PCB 118	76.4 ± 7.7	1.2 ± 0.4	32.8 ± 5.5	42%
					PCB 123	76.3 ± 10.2	0.4 ± 0.1	14.1 ± 4.4	62%
					PCB 126	**74.2 ± 11.1**	**31.3 ± 7.7**	**21.0 ± 4.0**	22%
					PCB 156	**63.2 ± 15.5**	**4.3 ± 2.6**	**82.2 ± 29.6**	0
					PCB 157	**68.0 ± 11.4**	**3.2 ± 1.6**	**72.9 ± 22.2**	0
					PCB 167	73.4 ± 9.2	1.3 ± 0.3	20.1 ± 5.0	52%
					PCB 169	**64.4 ± 11.7**	**49.9 ± 11.2**	**38.9 ± 10.8**	0
					PCB 189	**66.0 ± 11.9**	**4.2 ± 1.7**	**62.7 ± 26.3**	0
mean ± std	52.3 ± 8.6	21.6 ± 7.3	7.3 ± 2.0	23%	mean ± std	75.1 ± 9.6	6.1 ± 1.7	34.9 ± 11.1	34%
WHO PCDD/F TEQ	70.7 ± 8.9	21.6 ± 8.8	8.1 ± 2.0	41%	WHO PCB TEQ	**72.0 ± 11.2**	**34.2 ± 8.9**	**23.9 ± 4.6**	14%

Bold values: bioavailability values basing on fecal endpoint were close to the sum of liver and fat endpoints.

Speculated metabolization ratio: equals bioavailability values of fecal endpoint minus the sum bioavailability values of liver and fat endpoints.

*Estimated whole SD rat body adipose tissue weight, multiplying body fat index (6.5%) by each rat’s weight.

In the present study, the calculation of the feces endpoint was based on the following assumption: after administration, the pollutants were either bioavailable or unavailable. If available, then they might be metabolized to daughter chemicals or distribute, equilibrate, and accumulate in depots such as liver or fat as parent pollutants. If unavailable, then they might be excreted via feces or urine. If we consider the rat body as a whole depot, then the total amount of pollutants administered minus the total parent pollutant amount in the excrement equals the bioavailable pollutant fraction. If we consider the liver and fat as the main depots, then fecal bioavailability should equal the sum of liver bioavailability and fat bioavailability. Taking 1,2,3,4,7,8-HxCDD as an example, the feces endpoint bioavailability of 1,2,3,4,7,8-HxCDD was 57.5 ± 15.4%, indicating that the bioavailable congener was approximately 57.5%, which was deduced from the percentage (42.5%) of the non-bioavailable parent 1,2,3,4,7,8-HxCDD detected in the feces. Moreover, the feces endpoint value (57.5%) was close to the sum of liver bioavailability (40.3%) and fat bioavailability (14.3%), that is, the feces endpoint results were validated by liver and fat endpoint results. However, as indicated in Table 1, we noticed that only the gray-shaded congener endpoints were generally consistent within each endpoint. For 2,3,7,8-TeCDF and PCB 28, the feces bioavailability was far greater than the sum of liver bioavailability and fat bioavailability, indicating that the feces bioavailability might have been overestimated. In such a case, the “missing” parent pollutants neither accumulated in the feces nor in liver or fat, and one possible explanation was that they might had been metabolized to daughter pollutants. Although in the present study we did not measure the metabolites of PCDD/F or PCB, their existence has been proved by previous toxicokinetic studies. For example, dihydroxy-TrCDD and dihydroxy-tetra chlorodiphenyl ether were reported as the main metabolites of 2,3,7,8-TCDD, monohydroxy-TCDF and dihydroxy-TrCDF were the major metabolites of 2,3,7,8-TCDF presenting in the rat bile^20–22^, and 4’-OH-PCB 178 was the major metabolite of PCB 187 found in rat’s feces^23^. Metabolic transformation depends on the chlorine substitution pattern in the molecule. Metabolic reactions include oxygen bridge cleavage, oxidation and reductive dechlorination, involving also an occurrence of aromatic methyl migration (NIH shift) from the lateral to the peri position^24^. The metabolic transformation of TCDD in the liver was believed to be a rate-limiting step in the elimination of TCDD from the body^25^. In this study, the speculated metabolization ratios of PCDD/F and PCB are summarized in Table 1.

An ideal endpoint of bioavailability should accurately and comprehensively reflect the accumulation of lipophilic pollutants with reliable reproducibility, and it is much better if the approach is friendly to experimental animals (ethical issues) and/or researchers (easy to handle). From a practical perspective, blood and liver (or other organs), which can accumulate pollutants, are relatively easy to obtain and comparable with existing values in the literature. For these advantages, blood and liver are common endpoints in bioavailability studies. For example, using rat blood as the endpoint and normalizing the gavage (corn oil) AUC to the intravenous injection AUC, Pu et al. measured the absolute bioavailability of PCB 52 (53–67%) and PCB 118 (61–70%)^26^. In another study, Fries et al. reported the bioavailability of PCB 52 (88%) and PCB 101(77%) using feces as the endpoint^27^. In the present study, we obtained relatively similar results (PCB 52: 87.0 ± 6.4%; PCB 101: 88.8 ± 5.0%; PCB 118: 76.4 ± 7.7%) when using feces as the endpoint (Table 1). However, when using liver or fat as the endpoint, PCB congener bioavailability varied significantly (0.1%–32.8%). As relevant research on bioavailability was limited, we were unable to make further comparisons for dioxin-like PCBs, including PCB 126, 156, and 169.

Therefore, to answer the question whether feces can be used as an indirect endpoint for the bioavailability measurement of POP, it depended on how you define the process of bioavailability. If bioavailability is defined as involving the metabolization (degradation) of compounds, then the fecal endpoint is a feasible option. However, if the selective accumulation behavior of some congeners in different organs/tissues is considered, regardless of the component (feces, liver, or fat), then there is no one comprehensive endpoint suitable for all congeners.

### PCDD/F and PCB bioavailability differences between male and female rats

Gender-specific mechanisms of POP bioavailability has not been widely studied yet. Evan et al. observed gender differences in the response of nonreproductive cells when adipose cells from males and females were exposed to 2,3,7,8-TCDD, e.g., 2,3,7,8-TCDD induced lipid peroxidation in the adipose tissues of male guinea pigs, while it had no effect in females^28^. In our previous study, we also observed that curcumin lowered the TEQ of DL-PCBs in the liver of male rats, but not female rats^29^. In the present study, we examined PCDD/F and PCB in rat feces, liver and fat respectively, to observe whether gender difference in bioavailability exists.

#### PCDD/F bioavailability

For the feces endpoint, the PCDD/F congener bioavailability gradually decreased from tetra-(77.4%) to octa-(13.3%), and female rats showed significantly (*p* < 0.01) higher bioavailability than male rats at low dose (Fig. 1A1,A3). For the liver endpoint, female rats also showed higher bioavailability than male rats. However, unlike the decreasing linear trend of the fecal endpoint, a selective bioaccumulatory trend in the liver was observed (Fig. 1B1–B4). For example, 1,2,3,4,7,8-HxCDF (50.6%) and 1,2,3,4,7,8-HxCDD (40.3%) were the dominate congeners. For the fat endpoint, the bioaccumulation of PCDD/F was lower than that in feces or liver, and no significant differences were observed between female and male rats in either dose groups. Moreover, an interesting phenomenon was observed in that PCDD (Fig. 1C3–C4), seemed to show higher accumulation in fat tissues than PCDF (Fig. 1C1–C2), although the difference was not significant. As far as the TEQ bioavailability of PCDD/F is concerned, as indicated in Table 2, for the feces endpoint, although the mean values of both female groups were higher than that of male groups, no significant differences were observed. For the liver endpoint, the mean TEQ bioavailability of female rats was higher than that of male rats, and the high dose group showed that the differences between male and female rats were significant (*p* < 0.05). For the fat endpoint, the mean bioavailability between male and female rats was similar, and no significant difference was observed.

**Table 2:** The TEQ bioavailability of PCDD/F & PCB calculated by different endpoints.

	Control		Low dose #		High dose #	
	♀, (*n* = 3)	♂, (*n* = 3)	♀, (*n* = 3)	♂, (*n* = 3)	♀, (*n* = 3)	♂, (*n* = 3)
Administered PCDD/F dose via polluted oil (ng, WHO-TEQ)	0.57 ± 0.06 ##	0.76 ± 0.01 ##	107 ± 7.1	140 ± 4.2	404 ± 12.2	619 ± 17.4
Output via feces (ng, WHO-TEQ_PCDD/F_)	0.12 ± 0.01	0.15 ± 0.01	21.1 ± 2.5	41.9 ± 10.0	113 ± 1.7	245 ± 64.6
Output via urine (ng, WHO-TEQ_PCDD/F_)	ND	ND	0.08 ± 0.04	0.08 ± 0.04	0.09 ± 0.02	0.07 ± 0.02
Bioavailability of PCDD/F (%), feces	78.4 ± 0.2	80.2 ± 1.9	80.1 ± 3.0	70.2 ± 6.8	72.0 ± 0.6	60.6 ± 9.5
Bioavailability of PCDD/F (%), liver	N/A	N/A	23.0 ± 3.9	13.0 ± 1.4	33.7 ± 2.5	16.2 ± 2.7*
Bioavailability of PCDD/F (%), fat	N/A	N/A	20.2 ± 3.1	21.1 ± 1.0	13.8 ± 1.5	13.0 ± 0.8*
Administered PCB dose via polluted oil (ng, WHO-TEQ)	0.1 ± 0.01 ##	0.1 ± 0.02 ##	40.7 ± 2.7	53.5 ± 1.6	149 ± 4.5	228 ± 6.4
Output via feces (ng, WHO-TEQ_PCB_)	0.018 ± 0.0	0.026 ± 0.01	6.6 ± 1.1	11.7 ± 5.3	42.7 ± 2.5	69.5 ± 30.4
Output via urine (ng, WHO-TEQ_PCB_)	ND	ND	0.02 ± 0.01	0.02 ± 0.01	0.06 ± 0.01	0.05 ± 0.01
Bioavailability of PCB (%), feces	82.7 ± 1.4	79.0 ± 5.2	83.8 ± 3.2	74.2 ± 7.4	71.3 ± 1.6	58.7 ± 11.8
Bioavailability of PCB (%), liver	N/A	N/A	44.0 ± 6.8	26.0 ± 2.7	33.4 ± 4.2	38.5 ± 3.3
Bioavailability of PCB (%), fat	N/A	N/A	16.3 ± 2.5	17.8 ± 1.4	14.2 ± 2.7	13.9 ± 4.3

#For PCDD/F: Low dose: 0.2 ng/100 g b.w./d; High dose: 1 ng/100 g b.w./d; For PCB: Low dose: 1 ng/100 g b.w./d; High dose: 5 ng/100 g b.w./d.

##For control group, SD rats were administrated with blank oil containing negligible PCDD/F or PCB. Values showed in the table were background level pre-existed in feeds.

*ND* Not detected; *N*/*A* Not applicable.

*significant difference comparison between female and male, *t*-test, type1, tail 2, *p* < 0.05.

It is reported that the toxicity of PCDD/Fs is not produced by the metabolite but the parent compound. Thus, metabolism and excretion of these compounds mainly represent a detoxification process^24^. In an extensive review on the toxicokinetics and metabolism of PCDD/F, Van den Berg et al. summarized that the body distribution of PCDD/F was strongly species dependent. For rodents and mammals, liver and adipose tissue were the major storage sites. In addition, the high liver retention observed for 2,3,7,8-substituted PCDD/Fs in rodents has been attributed to the presence of specific and inducible storage sites, e.g. CYPlA2 in the liver cell^24^. In general, these conclusions give clues to explain our findings.

#### PCB bioavailability

Similar to PCDD/Fs, for the feces endpoint, PCB bioavailability (63.2–88.8%) generally decreased from tri- to hepta-chlorinated congeners, and female rats showed significantly higher bioavailability than male rats in both low (*p* < 0.01) and high (*p* < 0.01) dose groups (Fig. 2A1–A2). It was interesting to observe specific accumulation for PCB 169 (49.9%) and PCB 126 (31.3%) in the liver, but the other congeners were generally detected at low levels (0.0–5.1%), and no significant difference between male and female rats was observed (Fig. 2B1–B2). Unlike the liver, fat tissues are one of the main depots for PCB (mean bioavailability: 34.9%). In the high dose group, female rats showed a significantly higher bioavailability than male rats (Fig. 2C2, *p* < 0.01). In terms of the TEQ bioavailability, the mean TEQ bioavailability of PCB for feces, liver, and fat endpoints were 72.0%, 34.2% and 23.9% respectively, and no significant differences were observed in these three endpoints (Table 2). PCBs with fewer chlorine atoms are less persistent and more susceptible to metabolic attack. In animals and plants, the metabolites of PCBs are mainly hydroxylated PCBs (OH-PCBs). This biotransformation is primarily carried out by cytochrome P-450-dependent monooxygenases^30^. This might possibly explain why the lower chlorinated PCB congeners bioavailability basing on liver and fat endpoint was lower than those higher chlorinated congeners.

### Limitation of this study

First, it was difficult to obtain the exact mass of rat organs such as blood, muscle, fat, and feces (usually mixed with feed), and the rat’s body fat ratio in the present study was set to 6.5% according to a previous study^31^. Furthermore, we did not measure the skin, bone, and hair of the rats, although the accumulation of POPs in these tissues was expected to be very low. Therefore, there was a deviation in the bioavailability based on the mass of organs. Second, we did not measure the metabolites of PCDD/F or PCB in the present study, their existence was speculated by previous toxicokinetic studies. In future study, the measurement of PCDD/F or PCB metabolites should be taken into account. Third, the accumulation and excretion of pollutants is a dynamic process, so the duration of the experiment might have directly impacted the bioavailability results.

## Conclusions

In this study, we examined the accumulation and distribution of 10 PCDD/F and 18 PCB congeners in feces, urine, liver, and fat of SD rats, and calculated the bioavailability based on feces, liver, and fat endpoints. Our results indicated that PCB 126, PCB 169, and 50% of PCDD/F congeners were mainly accumulated in the liver, with a bioavailability of 28.9–50.6%. On the other hand, higher chlorinated (> 5 Cl) PCB congeners were mainly accumulated in fat tissues, with a bioavailability of 20.1–82.2%, while lower chlorinated (< 5 Cl) pollutants, such as 2,3,7,8-TeCDF, 2,3,7,8-TeCDD, 1,2,3,7,8-PeCDF, and PCB 28, 52, 77, 81, were likely metabolized over 36% in rats during the 8-week experimental period. If we considered metabolization (degradation) as a type of bioavailable process, then the fecal endpoint was a feasible option. However, if we did not consider so, regarding the selective accumulation behavior of some congeners in different organs/tissues, then the fecal endpoint was not a complete option. Based on our observations, female rats showed significantly higher PCDD/F bioavailability than male rats at low dose level (0.2 ng/100 g b.w./d); however, the difference in PCB bioavailability between female and male rats was not significant.

## Supplementary Information

Supplementary Information.

## Data Availability

The datasets used and/or analyzed during the current study available from the corresponding author on reasonable request.
