# Supplementary material for: Use of different endpoints to determine the bioavailability of polychlorinated dibenzo-p-dioxins/furans (PCDD/Fs) and polychlorinated biphenyls (PCBs) in Sprague–Dawley rats
Source: Sci Rep. 2022 Nov 28;12:20433. doi: 10.1038/s41598-022-25042-3 (PMC9705297; doi:10.1038/s41598-022-25042-3)
Supplement: Supplementary file 1 — Supplementary Information. [file 41598_2022_25042_MOESM1_ESM.docx]

Table S1. Measured concentrations of PCDD/F and PCBs in gavage soybean oil (low and high level) and rodent feed.

|  | | Oil/blank (ng/mL) | Oil/Low  (ng/mL) | Oil/High  (ng/mL) | Feed/background level (ng/g) |
| --- | --- | --- | --- | --- | --- |
| No. |  | Non-spiked | Spiked | Spiked | Non-spiked |
|  | PCDD/F Congeners | | | | |
| 1 | 2,3,7,8-TeCDF | ND | 1.292 | 6.228 | 0.000051 |
| 2 | 1,2,3,7,8-PeCDF | ND | 1.314 | 6.177 | 0.000062 |
| 3 | 1,2,3,4,7,8-HxCDF | ND | 1.256 | 6.152 | 0.000097 |
| 4 | 1,2,3,4,6,7,8-HpCDF | ND | 0.983 | 4.862 | 0.000337 |
| 5 | OCDF | ND | 1.407 | 6.629 | 0.003236 |
| 6 | 2,3,7,8-TeCDD | ND | 2.016 | 8.112 | 0.000016 |
| 7 | 1,2,3,7,8-PeCDD | ND | 1.549 | 6.319 | 0.000049 |
| 8 | 1,2,3,4,7,8-HxCDD | ND | 1.380 | 5.705 | 0.000929 |
| 9 | 1,2,3,4,6,7,8-HpCDD | ND | 1.471 | 6.705 | 0.001257 |
| 10 | OCDD | ND | 1.335 | 6.586 | 0.003667 |
|  | Total PCDD/Fs | ND | 14.00 | 63.48 | 0.0097 |
| No. | PCBs Congeners | | | | |
| 1 | PCB 28 | 0.01308 | 15.490 | 44.757 | 0.01046 |
| 2 | PCB 52 | 0.01241 | 31.011 | 67.363 | 0.02348 |
| 3 | PCB 101 | 0.00611 | 22.302 | 72.748 | 0.01505 |
| 4 | PCB 138 | 0.00638 | 14.941 | 51.534 | 0.00984 |
| 5 | PCB 153 | 0.01033 | 16.074 | 58.167 | 0.01389 |
| 6 | PCB 180 | 0.00263 | 14.701 | 60.034 | 0.00427 |
| 7 | PCB 77 | 0.000887 | 20.444 | 56.561 | 0.00302 |
| 8 | PCB 81 | ND | 10.977 | 42.552 | 0.00049 |
| 9 | PCB 105 | 0.003722 | 12.771 | 53.386 | 0.0038 |
| 10 | PCB 114 | ND | 13.096 | 51.124 | 0.00069 |
| 11 | PCB 118 | 0.007356 | 26.393 | 106.760 | 0.00916 |
| 12 | PCB 123 | 0.000669 | 11.668 | 48.531 | 0.00059 |
| 13 | PCB 126 | 0.000514 | 12.170 | 48.185 | 0.00064 |
| 14 | PCB 156 | 0.000798 | 11.779 | 50.192 | 0.00171 |
| 15 | PCB 157 | 0.000119 | 12.906 | 50.384 | 0.00086 |
| 16 | PCB 167 | 0.000699 | 13.371 | 53.013 | 0.00271 |
| 17 | PCB 169 | 0.001316 | 11.850 | 48.806 | 0.00052 |
| 18 | PCB 189 | 0.000148 | 12.845 | 48.990 | 0.00065 |
|  | Total PCBs | 0.0672 | 284.8 | 1013 | 0.1018 |
| ND: not detected | | | | | |

Table S2. Sample information (volumes/grams)

| Volumes/grams | | Oil/blank  administrated (mL) | Oil/Low  administrated (mL) | Oil/High  administrated (mL) | Feed  (g) | Liver  (g) | Fat *  (g) | Fat ^#^  (g) | muscle  (g) | Other  organs | Blood  (mL) | Feces  (g d.w.) | urine  (mL) | Fasted b.w (g) @ sacrifice day |
| --- | --- | --- | --- | --- | --- | --- | --- | --- | --- | --- | --- | --- | --- | --- |
| Control  group | **♀1** | 23.5 | / | / | 1278 | 6.7 | 7.8 | 16.5 | 30.6 | 30.1 | 2.5 | 460 | 600 | 253 |
|  | **♀2** | 26.7 | / | / | 1424 | 8.3 | 6.6 | 19.1 | 33.3 | 35.1 | 5 | 460 | 1000 | 293 |
|  | **♀3** | 23.1 | / | / | 1152 | 7.1 | 4.9 | 15.6 | 28.2 | 25.0 | 6.5 | 360 | 950 | 240 |
|  | **♂1** | 34.2 | / | / | 1700 | 13.1 | 5.6 | 26.3 | 37.1 | 35.8 | 4 | 630 | 900 | 405 |
|  | **♂2** | 35.7 | / | / | 1697 | 14 | 1.8 | 28.4 | 55.5 | 47.1 | 11 | 530 | 680 | 437 |
|  | **♂3** | 36.1 | / | / | 1732 | 12 | 7.2 | 28.3 | 37.3 | 39.8 | 29.4 | 530 | 650 | 436 |
| Low dosage  Group | **♀1** | / | 27.7 | / | 1433 | 8.9 | 5.3 | 16.4 | 29.4 | 30.1 | 2 | 510 | 390 | 252 |
|  | **♀2** | / | 24.5 | / | 1266 | 8.1 | 12.5 | 17.2 | 30.5 | 32.6 | 4.5 | 380 | 300 | 265 |
|  | **♀3** | / | 25.1 | / | 1229 | 9.2 | 3.4 | 17.8 | 35.1 | 35.5 | 3.5 | 340 | 1400 | 274 |
|  | **♂1** | / | 32.9 | / | 1596 | 13.8 | 3.5 | 25.8 | 45.4 | 40.1 | 10 | 450 | 710 | 397 |
|  | **♂2** | / | 33.7 | / | 1692 | 13.8 | 7.8 | 27.2 | 32.9 | 29.7 | 4.5 | 520 | 900 | 419 |
|  | **♂3** | / | 34.9 | / | 1671 | 14.3 | 5.2 | 27.9 | 44.4 | 40.1 | 7 | 530 | 410 | 429 |
| High  Dosage  group | **♀1** | / | / | 24.3 | 1261 | 8.1 | 3.6 | 17.4 | 33.9 | 38.7 | 4.5 | 410 | 590 | 268 |
|  | **♀2** | / | / | 23.4 | 1141 | 8.1 | 2.9 | 16.2 | 33 | 30.2 | 3 | 358 | 1050 | 249 |
|  | **♀3** | / | / | 22.9 | 1174 | 7.4 | 5.4 | 15.1 | 33.9 | 29.5 | 5.5 | 365 | 700 | 233 |
|  | **♂1** | / | / | 36.6 | 1676 | 17.4 | 9.1 | 30.4 | 36.8 | 28.4 | 6 | 545 | 800 | 467 |
|  | **♂2** | / | / | 34.9 | 1672 | 15.1 | 5.5 | 28.4 | 51.7 | 41.5 | 9.5 | 500 | 1200 | 436 |
|  | **♂3** | / | / | 36.7 | 1740 | 13.8 | 6.5 | 29.4 | 55.6 | 45.0 | 12.5 | 556 | 570 | 453 |

Fat*: White adipose tissue collected near gonad.

Fat ^#^: Whole SD rat body adipose tissue, estimated by multiply body fat index (6.5%) by each rat’s body weight (Schoeffner et al., Organ weights and fat volume in rats as a function of strain and age. Journal of Toxicology and Environmental Health-Part A 1999; 56: 449-462);

Other organs: mixture of kidney, pancreas, testes or ovaries, and heart.

Table S3-1. Summary of PCDD/F and PCB congener bioavailabilities in each endpoint (feces).

| **Feces** | | | | | |
| --- | --- | --- | --- | --- | --- |
| No. |  | low♀ | low♂ | high♀ | high♂ |
|  | PCDD/F Congeners | | | | |
| 1 | 2,3,7,8-TeCDF | 86.13 | 74.85 | 80.66 | 68.07 |
| 2 | 2,3,7,8-TeCDD | 84.07 | 74.87 | 77.51 | 66.27 |
| 3 | 1,2,3,7,8-PeCDF | 76.65 | 65.50 | 70.02 | 58.03 |
| 4 | 1,2,3,7,8-PeCDD | 78.76 | 68.25 | 69.26 | 56.84 |
| 5 | 1,2,3,4,7,8-HxCDF | 65.01 | 53.33 | 55.85 | 48.40 |
| 6 | 1,2,3,4,7,8-HxCDD | 67.63 | 57.39 | 58.36 | 61.22 |
| 7 | 1,2,3,4,6,7,8-HpCDF | 39.80 | 30.79 | 31.02 | 27.90 |
| 8 | 1,2,3,4,6,7,8-HpCDD | 47.31 | 38.31 | 37.87 | 33.08 |
| 9 | OCDF | 42.66 | 36.42 | 22.90 | 26.70 |
| 10 | OCDD | 15.33 | 5.72 | 16.78 | 15.53 |
|  | PCBs Congeners | | | | |
| 1 | PCB 28 | 87.1 | 82.9 | 79.9 | 67.0 |
| 2 | PCB 52 | 93.8 | 88.8 | 87.0 | 78.6 |
| 3 | PCB 77 | 93.3 | 87.9 | 82.8 | 73.5 |
| 4 | PCB 81 | 89.0 | 81.4 | 80.2 | 68.7 |
| 5 | PCB 101 | 93.8 | 90.5 | 88.3 | 82.7 |
| 6 | PCB 123 | 86.8 | 78.2 | 76.4 | 63.9 |
| 7 | PCB 126 | 85.8 | 77.2 | 73.0 | 60.9 |
| 8 | PCB 114 | 85.5 | 77.6 | 75.6 | 66.3 |
| 9 | PCB 105 | 84.8 | 76.7 | 74.9 | 64.0 |
| 10 | PCB 118 | 83.8 | 75.5 | 78.3 | 68.1 |
| 11 | PCB 167 | 83.1 | 72.2 | 74.8 | 63.6 |
| 12 | PCB 138 | 84.1 | 79.4 | 77.2 | 73.9 |
| 13 | PCB 156 | 79.7 | 69.2 | 59.8 | 44.1 |
| 14 | PCB 157 | 80.6 | 70.1 | 66.3 | 54.8 |
| 15 | PCB 169 | 76.8 | 63.7 | 65.6 | 51.5 |
| 16 | PCB 153 | 79.7 | 68.4 | 75.3 | 66.4 |
| 17 | PCB 189 | 78.3 | 70.1 | 63.5 | 52.0 |
| 18 | PCB 180 | 75.2 | 62.0 | 74.2 | 64.8 |

Table S3-2. Summary of PCDD/F and PCB congener bioavailabilities in each endpoint (liver).

| **Liver** | | | | | |
| --- | --- | --- | --- | --- | --- |
| No. |  | low♀ | low♂ | high♀ | high♂ |
|  | PCDD/F Congeners | | | | |
| 1 | 2,3,7,8-TeCDF | 2.8 | 2.0 | 2.8 | 1.7 |
| 2 | 2,3,7,8-TeCDD | 11.0 | 6.3 | 16.7 | 8.0 |
| 3 | 1,2,3,7,8-PeCDF | 3.8 | 2.1 | 3.8 | 2.2 |
| 4 | 1,2,3,7,8-PeCDD | 33.1 | 17.9 | 51.2 | 22.9 |
| 5 | 1,2,3,4,7,8-HxCDF | 58.6 | 37.4 | 70.3 | 36.3 |
| 6 | 1,2,3,4,7,8-HxCDD | 42.7 | 23.8 | 61.0 | 33.6 |
| 7 | 1,2,3,4,6,7,8-HpCDF | 30.8 | 23.2 | 39.4 | 23.6 |
| 8 | 1,2,3,4,6,7,8-HpCDD | 32.5 | 21.2 | 39.2 | 22.7 |
| 9 | OCDF | 8.9 | 6.4 | 8.5 | 5.8 |
| 10 | OCDD | 14.5 | 10.2 | 15.4 | 9.6 |
|  | PCBs Congeners | | | | |
| 1 | PCB 28 | 0.1 | 0.2 | 0.1 | 0.1 |
| 2 | PCB 52 | 0.1 | 0.1 | 0.1 | 0.1 |
| 3 | PCB 77 | 0.0 | 0.0 | 0.1 | 0.0 |
| 4 | PCB 81 | 6.1 | 3.0 | 7.5 | 3.7 |
| 5 | PCB 101 | 0.1 | 0.1 | 0.0 | 0.0 |
| 6 | PCB 123 | 0.6 | 0.4 | 0.4 | 0.3 |
| 7 | PCB 126 | 40.4 | 22.7 | 29.2 | 33.1 |
| 8 | PCB 114 | 1.3 | 1.1 | 1.9 | 1.1 |
| 9 | PCB 105 | 0.8 | 0.7 | 0.6 | 0.6 |
| 10 | PCB 118 | 1.1 | 1.3 | 1.4 | 0.9 |
| 11 | PCB 167 | 1.4 | 1.1 | 1.7 | 1.0 |
| 12 | PCB 138 | 1.2 | 1.7 | 3.0 | 2.1 |
| 13 | PCB 156 | 2.4 | 2.1 | 8.2 | 4.4 |
| 14 | PCB 157 | 2.1 | 2.1 | 5.6 | 3.2 |
| 15 | PCB 169 | 57.2 | 37.8 | 47.5 | 57.2 |
| 16 | PCB 153 | 1.3 | 1.9 | 2.8 | 1.9 |
| 17 | PCB 189 | 3.9 | 2.9 | 3.8 | 6.1 |
| 18 | PCB 180 | 1.6 | 2.6 | 3.1 | 2.3 |

Table S3-3. Summary of PCDD/F and PCB congener bioavailabilities in each endpoint (fat).

| **Fat** | | | | | |
| --- | --- | --- | --- | --- | --- |
| No. |  | low♀ | low♂ | high♀ | high♂ |
|  | PCDD/F Congeners | | | | |
| 1 | 2,3,7,8-TeCDF | 0.8 | 1.0 | 0.4 | 0.6 |
| 2 | 2,3,7,8-TeCDD | 6.2 | 6.7 | 4.4 | 4.2 |
| 3 | 1,2,3,7,8-PeCDF | 0.9 | 1.0 | 0.6 | 0.7 |
| 4 | 1,2,3,7,8-PeCDD | 7.8 | 7.7 | 5.2 | 4.8 |
| 5 | 1,2,3,4,7,8-HxCDF | 2.1 | 2.9 | 1.7 | 1.4 |
| 6 | 1,2,3,4,7,8-HxCDD | 5.1 | 5.7 | 3.5 | 3.4 |
| 7 | 1,2,3,4,6,7,8-HpCDF | 1.0 | 1.2 | 0.7 | 0.7 |
| 8 | 1,2,3,4,6,7,8-HpCDD | 1.6 | 1.6 | 1.0 | 1.0 |
| 9 | OCDF | 0.3 | 0.4 | 0.1 | 0.1 |
| 10 | OCDD | 0.6 | 0.8 | 0.3 | 0.3 |
|  | PCBs Congeners | | | | |
| 1 | PCB 28 | 1.2 | 2.2 | 1.9 | 1.6 |
| 2 | PCB 52 | 0.8 | 1.3 | 2.5 | 2.2 |
| 3 | PCB 77 | 0.2 | 0.3 | 0.2 | 0.3 |
| 4 | PCB 81 | 4.6 | 4.3 | 2.5 | 2.2 |
| 5 | PCB 101 | 1.8 | 1.3 | 1.3 | 1.5 |
| 6 | PCB 123 | 11.6 | 10.4 | 7.1 | 5.9 |
| 7 | PCB 126 | 14.3 | 15.1 | 11.8 | 10.9 |
| 8 | PCB 114 | 27.8 | 24.5 | 25.1 | 17.9 |
| 9 | PCB 105 | 23.1 | 18.0 | 16.5 | 11.7 |
| 10 | PCB 118 | 22.9 | 21.1 | 21.5 | 15.7 |
| 11 | PCB 167 | 14.7 | 15.3 | 11.2 | 8.7 |
| 12 | PCB 138 | 31.1 | 32.6 | 67.5 | 36.6 |
| 13 | PCB 156 | 42.0 | 38.2 | 77.9 | 45.8 |
| 14 | PCB 157 | 37.9 | 39.6 | 65.0 | 38.3 |
| 15 | PCB 169 | 23.1 | 27.5 | 21.9 | 24.1 |
| 16 | PCB 153 | 35.2 | 38.3 | 67.2 | 40.4 |
| 17 | PCB 189 | 32.8 | 25.9 | 63.8 | 32.8 |
| 18 | PCB 180 | 32.7 | 35.0 | 55.4 | 30.7 |

|  | |  | **PCDD/F** | | | | | |  | **PCB** | | | | | | |
| --- | --- | --- | --- | --- | --- | --- | --- | --- | --- | --- | --- | --- | --- | --- | --- | --- |
| Bioavailability  (%) | | Feces | Liver | Fat | Blood | Muscle | Urine | Other organs * |  | Feces | Liver | Fat | Blood | Muscle | Urine | Other organs |
| Low dosage  Group | **♀1** | 82.7 | 18.6 | 16.7 | N/A | 0.33 | <0.01 | 0.83 |  | 86.4 | 36.3 | 44.0 | 0.03 | 0.82 | <0.01 | 4.30 |
|  | **♀2** | 80.8 | 24.1 | 21.4 | N/A | 0.31 | <0.01 | 1.63 |  | 84.7 | 49.3 | 53.7 | 0.06 | 1.08 | <0.01 | 8.88 |
|  | **♀3** | 76.9 | 26.2 | 22.6 | N/A | 0.39 | <0.01 | 1.10 |  | 80.1 | 46.3 | 60.1 | 0.02 | 1.07 | <0.01 | 7.91 |
|  | **♂1** | 77.3 | 14.3 | 21.6 | N/A | 0.29 | <0.01 | 0.33 |  | 81.7 | 28.9 | 54.0 | 0.03 | 7.01 | <0.01 | 1.75 |
|  | **♂2** | 63.7 | 13.2 | 20.0 | N/A | 0.21 | <0.01 | 0.11 |  | 66.8 | 25.3 | 56.3 | 0.02 | 0.91 | <0.01 | 0.58 |
|  | **♂3** | 69.6 | 11.6 | 21.9 | N/A | 0.15 | <0.01 | 0.42 |  | 74.0 | 23.7 | 62.6 | 0.04 | 0.62 | <0.01 | 1.78 |
| High  Dosage  group | **♀1** | 72.5 | 34.4 | 14.3 | N/A | 0.32 | <0.01 | 0.90 |  | 71.0 | 91.0 | 47.0 | 0.01 | 2.93 | <0.01 | 9.23 |
|  | **♀2** | 72.3 | 30.9 | 14.9 | N/A | 0.22 | <0.01 | 0.47 |  | 73.0 | 80.4 | 53.6 | 0.01 | 1.73 | <0.01 | 5.65 |
|  | **♀3** | 71.3 | 35.7 | 12.1 | N/A | 0.24 | <0.01 | 1.14 |  | 69.8 | 71.8 | 36.6 | 0.04 | 1.20 | <0.01 | 9.82 |
|  | **♂1** | 57.4 | 16.7 | 13.9 | N/A | 0.48 | <0.01 | 0.39 |  | 55.4 | 41.2 | 60.8 | 0.02 | 2.14 | <0.01 | 2.18 |
|  | **♂2** | 71.3 | 18.6 | 12.4 | N/A | 0.25 | <0.01 | 0.93 |  | 71.8 | 39.5 | 39.8 | 0.02 | 1.44 | <0.01 | 11.43 |
|  | **♂3** | 53.1 | 13.2 | 12.5 | N/A | 0.08 | <0.01 | 0.39 |  | 49.0 | 34.8 | 34.4 | 0.02 | 0.51 | <0.01 | 3.38 |
| Average |  | *70.7* | *21.5* | *17.0* | *N/A* | *0.27* | *<0.01* | *0.72* |  | *72.0* | *47.4* | *50.2* | *0.03* | *1.79* | *<0.01* | *5.58* |

Table S4. Comparison of PCDD/F/PCB bioavailabilities (mean values) obtained by different endpoints

*Other organs: mixture of kidney, pancreas, testes or ovaries, and heart

N/A, not applicable
